# Association of Cannabis Retailer Proximity and Density With Cannabis Use Among Pregnant Women in Northern California After Legalization of Cannabis for Recreational Use

**DOI:** 10.1001/jamanetworkopen.2021.0694

**Published:** 2021-03-04

**Authors:** Kelly C. Young-Wolff, Sara R. Adams, Alisa Padon, Lynn D. Silver, Stacey E. Alexeeff, Stephen K. Van Den Eeden, Lyndsay A. Avalos

**Affiliations:** 1 Division of Research, Kaiser Permanente Northern California, Oakland; 2 Public Health Institute, Oakland, California

## Abstract

This cross-sectional study examines the association of cannabis retailer proximity and density with cannabis use among pregnant women after legalization of cannabis for recreational use in California.

## Introduction

Prenatal cannabis use is associated with adverse perinatal outcomes^1^ and is increasing with expanding legalization.^2,3^ While it is known that retail availability of cannabis is associated with adult cannabis use,^4,5^ it is less clear whether living closer to a cannabis retailer or in a neighborhood with greater cannabis retailer density is associated with prenatal cannabis use. This cross-sectional study assessed the association between recreational use cannabis retailer availability and cannabis use among pregnant women served by the Kaiser Permanente Northern California (KPNC) heath system during the first year of California’s 2018 initiation of recreational cannabis sales.

## Methods

The KPNC institutional review board approved this and determined it to be exempt under Common Rule 45 CFR 46.104, as this study involves secondary research of identifiable private information for which consent is not required. The study followed the Strengthening the Reporting of Observational Studies in Epidemiology (STROBE) reporting guideline for cross-sectional studies.

KPNC serves more than 4 million patients and universally screens pregnant women for prenatal cannabis use through self-report and urine toxicological testing at entrance to prenatal care. All 39 278 women in KPNC’s 35-county catchment area who became pregnant and were screened for self-reported prenatal cannabis use (at approximately 8 weeks gestation) in 2018 were eligible for this cross-sectional study; 3856 women (9.8%) without toxicological test results and 227 women (0.6%) missing address data were excluded. Race/ethnicity was self-reported.

Storefront cannabis retailer addresses and license dates (proxy for operating dates) were collected from the California Bureau of Cannabis Control database. Patient and retailer addresses were geocoded, and drive times between addresses were computed using ArcGIS Pro version 2.2.4 (Esri). For each woman’s address, we calculated proximity to and density of cannabis retailers operating in 2018 between her last menstrual period and cannabis use screening date. Additional methods are provided in the eAppendix in the Supplement.

Adjusted odds ratios (aORs) and 95% CIs of prenatal cannabis use (self-report or toxicological results) by retail availability metrics were estimated using logistic regression in SAS statistical software version 9.4 (SAS Institute). *P* values were 2-sided, and statistical significance was set at *P* = .05. Data were analyzed from April to November 2020.

## Results

The total sample of 35 195 women (mean [SD] age, 30.9 [5.3] years) included 12 711 non-Hispanic white women (36.1%), 9472 Asian or Pacific Islander women (26.9%), 9329 Hispanic women (26.5%), and 2185 Black women (6.2%). A total of 4337 women (12.3%) were younger than 25 years, and 2839 women (8.1%) self-reported or had toxicological test results positive for prenatal cannabis use (Table). Women who used cannabis were younger, more likely to be Black, and less likely to be Asian or Pacific Islander. They lived in areas with greater neighborhood deprivation and underwent prenatal screening later in pregnancy. There were 208 qualifying cannabis retailers; women had a mean (SD) drive time to the nearest retailer of 16.0 (12.5) minutes and a mean (SD) of 4.0 (5.5) retailers within a 15-minute drive from their homes. The median (interquartile range) drive time to the nearest retailer was 12.5 (7.0-22.0) minutes, and the median (interquartile range) number of retailers within a 15-minute drive was 2.0 (0.0-6.0).

**Table.** Characteristics of Pregnant Women in Kaiser Permanente Northern California (KPNC) in 2018, Overall and by Prenatal Cannabis Use

Characteristic	Total, No. (%) (N = 35 195)	Prenatal Cannabis Use		
		Yes, No. (%) (n = 2839)	No, No. (%) (n = 32 356)	*P* value
Age, y				
Mean (SD)	30.9 (5.3)	27.6 (5.7)	31.1 (5.1)	<.001
≤24	4337 (12.3)	936 (33.0)	3401 (10.5)	<.001
25-34	22 090 (62.8)	1524 (53.7)	20 566 (63.6)	
>34	8768 (24.9)	379 (13.3)	8389 (25.9)	
Race/ethnicity				
Hispanic	9329 (26.5)	819 (28.8)	8510 (26.3)	<.001
Black	2185 (6.2)	581 (20.5)	1604 (5.0)	
Asian/Pacific Islander	9472 (26.9)	194 (6.8)	9278 (28.7)	
Non-Hispanic White	12 711 (36.1)	1096 (38.6)	11 615 (35.9)	
Other, >1 race, or unknown	1498 (4.3)	149 (5.2)	1349 (4.2)	
Neighborhood Deprivation Index				
Quartile 1 (least deprived)	8746 (24.9)	404 (14.2)	8342 (25.8)	<.001
Quartile 2	8757 (24.9)	579 (20.4)	8178 (25.3)	
Quartile 3	8759 (24.9)	769 (27.1)	7990 (24.7)	
Quartile 4 (most deprived)	8749 (24.9)	1050 (37.0)	7699 (23.8)	
Missing	184 (0.5)	37 (1.3)	147 (0.5)	
Calendar quarter of screening				
Q1	3990 (11.3)	310 (10.9)	3680 (11.4)	.59
Q2	10 069 (28.6)	828 (29.2)	9241 (28.6)	
Q3	10 545 (30.0)	828 (29.2)	9717 (30.0)	
Q4	10 591 (30.1)	873 (30.8)	9718 (30.0)	
Trimester screened				
First	33 249 (94.5)	2619 (92.3)	30 630 (94.7)	<.001
Second	1663 (4.7)	186 (6.6)	1477 (4.6)	
Third	283 (0.8)	34 (1.2)	249 (0.8)	
Residence in urban area				
Yes	33 933 (96.4)	2746 (96.7)	31 187 (96.4)	.35
No	1262 (3.6)	93 (3.3)	1169 (3.6)	
Drive time to nearest cannabis retailer, min				
Mean (SD)	16.0 (12.5)	15.0 (12.7)	16.1 (12.5)	<.001
<5	5266 (15.0)	506 (17.8)	4760 (14.7)	<.001
5-9	9082 (25.8)	812 (28.6)	8270 (25.6)	
10-19	10 437 (29.7)	807 (28.4)	9630 (29.8)	
≥20	10 410 (29.6)	714 (25.1)	9696 (30.0)	
Cannabis retailers within ≤15-min drive, No.				
Mean (SD)	4.0 (5.5)	4.7 (5.8)	4.0 (5.5)	<.001
0	14 733 (41.9)	1025 (36.1)	13 708 (42.4)	<.001
1-2	4692 (13.3)	397 (14.0)	4295 (13.3)	
3-5	5723 (16.3)	460 (16.2)	5263 (16.3)	
≥6	10 047 (28.5)	957 (33.7)	9090 (28.1)	

Longer drive time to the nearest retailer was associated with lower odds of cannabis use (aOR per additional 5-minute drive time, 0.96 [95% CI, 0.95-0.98]; *P* < .001; aOR for ≥20-minute drive vs <5 minutes, 0.78 [95% CI, 0.69-0.88]; *P* < .001) (Figure). Similarly, having more retailers within a 15-minute drive was associated with greater odds of cannabis use compared with not living within a 15-minute drive of a retailer (aOR per additional retailer, 1.02 [95% CI, 1.01-1.02]; *P* < .001; aOR for 1-2 retailers, 1.16 [95% CI, 1.02-1.32]; *P* = .02; aOR for 3-5 retailers, 1.20 [95% CI, 1.06-1.35]; *P* = .004; aOR for ≥6 retailers, 1.29 [95% CI, 1.17-1.42]; *P* < .001).

**Figure.**
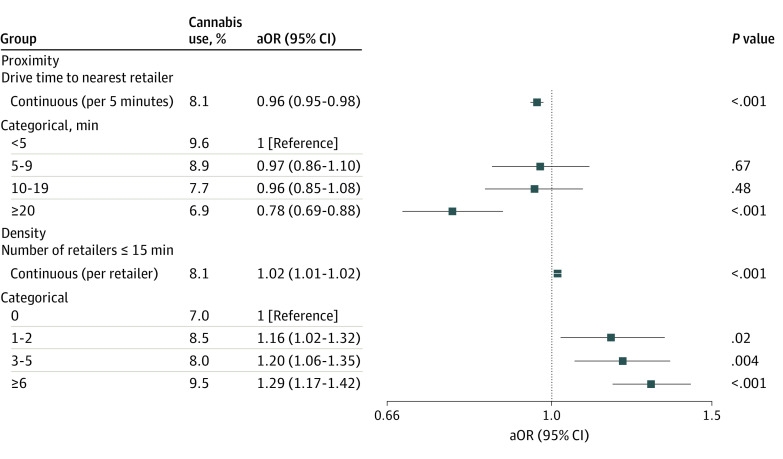
Adjusted Odds Ratios (aOR) with 95% CIs for Prenatal Cannabis Use by Proximity to and Density of Cannabis Retailers

## Discussion

This cross-sectional study found that, after the state-level legalization of cannabis for recreational use in California, greater retail availability was associated with higher odds of cannabis use among pregnant women; these results were consistent with a dose-response association. While easier access or greater exposure to storefront retailers may explain the association, research is needed to determine the direction of association, as retailers may be more likely to open in communities more receptive to cannabis.

This study has some limitations. Our sample was mostly limited to women screened in early pregnancy, and we were unable to evaluate cannabis use throughout pregnancy; in addition, while unlikely, some toxicological tests may have identified prepregnancy cannabis use. Additionally, only licensed cannabis retailers were included. Dates of licensure and operation may differ, and users may not purchase cannabis from a licensed retailer. Furthermore, all women in the sample received prenatal care in KPNC, and findings may not generalize to women without health insurance.

Our study took place during the first year of recreational cannabis legalization in California, when retailer access varied substantially. Despite potential harms,^1^ retailers frequently recommend cannabis for prenatal use,^6^ package warnings against use in pregnancy typically appear in the required minimum 6-point font, and in-store warnings about prenatal use are generally not required in California. As additional states legalize cannabis, there is an urgent need to understand the association between retail environments and prenatal cannabis use to inform policy and protect public health.

## Notes

Supplement. **eAppendix.** Supplemental Methods
