# Supplementary material for: Association of Cannabis Retailer Proximity and Density With Cannabis Use Among Pregnant Women in Northern California After Legalization of Cannabis for Recreational Use
Source: JAMA Netw Open. 2021 Mar 4;4(3):e210694. doi: 10.1001/jamanetworkopen.2021.0694 (PMC7933995; doi:10.1001/jamanetworkopen.2021.0694)
Supplement: Supplement. — eAppendix. Supplemental Methods [file jamanetwopen-e210694-s001.pdf]

Supplemental Online Content

Young-Wolff KC, Adams SR, Padon A, et al. Association of cannabis retailer proximity and density with cannabis use among pregnant women in Northern California after legalization of cannabis for recreational use. *JAMA Netw Open*. 2021;4(3):e210694. doi:10.1001/jamanetworkopen.2021.0694

eAppendix. Supplemental Methods

This supplemental material has been provided by the authors to give readers additional information about their work.

© 2021 Young-Wolff KC et al. *JAMA Network Open.*

eAppendix. Supplemental Methods

The first qualifying pregnancy was selected for 317 women with >1 pregnancy in 2018. Prenatal cannabis use was based on positive self-report of cannabis use during pregnancy and/or positive urine toxicology screening. Confirmatory testing for the presence of the cannabis metabolite, 11-nor-9-carboxy-delta 9- THC, was performed by liquid chromatography-tandem mass spectrometry for all positive immunoassay results. The confirmation test methodology was LC-MS/MS on a triple quadrupole system with a cutoff for positivity of 15ng/mL.

Our study included 208 adult-use cannabis retailers operating in our catchment area in 2018; 14 provided adult-use only and 194 provided adult-use and medical cannabis. Retailers ≤60 minutes of a patient's address were matched to each woman if they were open between her last menstrual period (calculated as 280 days prior to the estimated delivery date, which is determined by ultrasound for most patients) and screening date. Women with no adult-use retailer (n = 1,019) within 60 minutes were imputed to be 60 minutes to nearest retailer. The ArcGIS Pro OD Cost Matrix tool was used to calculate the proximity of participants' residences to the closest retailer, allowing us to determine the shortest travel distance (in drive time) along the street network. To determine retailer density, we calculated the count of geocoded retailers within a 15-minute drive (person-centered service area) in every direction from each woman's residence.

Analyses used logistic regression and adjusted for covariates that were significant in bivariate models, including age group, race/ethnicity, neighborhood deprivation index and trimester of prenatal substance use screening. The Neighborhood Deprivation Index (NDI) is a standardized measure of socioeconomic status by census track based on education, income and poverty, employment, housing, and occupation indicators in the 2018 American Community Survey of the U.S. Census Bureau.

© 2021 Young-Wolff KC et al. *JAMA Network Open.*
